# Machine learning approach for predicting post-intubation hemodynamic instability (PIHI) index values: towards enhanced perioperative anesthesia quality and safety

**DOI:** 10.1186/s12871-024-02523-8

**Published:** 2024-04-09

**Authors:** Rigele Te, Bo Zhu, Haobo Ma, Xiuhua Zhang, Shaohui Chen, Yuguang Huang, Geqi Qi

**Affiliations:** 1 Department of Anesthesiology, Peking Union Medical College Hospital, Chinese Academy of Medical Sciences & Peking Union Medical College, Beijing, 100730 China; 2 Department of Anesthesia, Critical Care and Pain Medicine, Beth Isreal Deaconess Medical Center, Boston, MA 02215 USA; 3 Key Laboratory of Transport Industry of Big Data Application Technologies for Comprehensive Transport, Beijing Jiaotong University, Beijing, 100044 China

**Keywords:** Perioperative anesthesia safety, Post-intubation hemodynamic instability, Drug infusion, Integrated coefficient of variation, Machine learning

## Abstract

**Background:**

Adequate preoperative evaluation of the post-intubation hemodynamic instability (PIHI) is crucial for accurate risk assessment and efficient anesthesia management. However, the incorporation of this evaluation within a predictive framework have been insufficiently addressed and executed. This study aims to developed a machine learning approach for preoperatively and precisely predicting the PIHI index values.

**Methods:**

In this retrospective study, the valid features were collected from 23,305 adult surgical patients at Peking Union Medical College Hospital between 2012 and 2020. Three hemodynamic response sequences including systolic pressure, diastolic pressure and heart rate, were utilized to design the post-intubation hemodynamic instability (PIHI) index by computing the integrated coefficient of variation (ICV) values. Different types of machine learning models were constructed to predict the ICV values, leveraging preoperative patient information and initiatory drug infusion. The models were trained and cross-validated based on balanced data using the SMOTETomek technique, and their performance was evaluated according to the mean absolute error (MAE), root mean square error (RMSE), mean absolute percentage error (MAPE) and R-squared index (R^2^).

**Results:**

The ICV values were proved to be consistent with the anesthetists’ ratings with Spearman correlation coefficient of 0.877 (*P* < 0.001), affirming its capability to effectively capture the PIHI variations. The extra tree regression model outperformed the other models in predicting the ICV values with the smallest MAE (0.0512, 95% CI: 0.0511–0.0513), RMSE (0.0792, 95% CI: 0.0790–0.0794), and MAPE (0.2086, 95% CI: 0.2077–0.2095) and the largest R^2^ (0.9047, 95% CI: 0.9043–0.9052). It was found that the features of age and preoperative hemodynamic status were the most important features for accurately predicting the ICV values.

**Conclusions:**

Our results demonstrate the potential of the machine learning approach in predicting PIHI index values, thereby preoperatively informing anesthetists the possible anesthetic risk and enabling the implementation of individualized and precise anesthesia interventions.

## Background

Anesthetic management is accompanied by high risks associated with perioperative complications [1]. In assessing these potential risks, there is growing concern regarding the preanesthetic evaluation, which is implemented via methods such as medical record analysis, patient interviews, physical examinations and preoperative tests [2]. Thus, designing effective and predictable evaluation indices to quantify the perioperative risk is of vital importance for improving the situational awareness [3] skills of the anesthetist and promoting safety management in anesthesiological practice [4] that relies on high levels of human performance [5].

Data-driven analysis and machine-learning models have become possible with increasingly structured data, such as preoperative electronic health/medical records [6]. Recently, various models have been developed to predict postoperative mortality [6, 7], postoperative pain [8], surgical acceptance [9], hypotension during anesthesia [10–12], surgery time [13, 14], intraoperative bradycardia [15], postoperative delirium [16, 17] and other risk outcomes [18–20]. Nevertheless, there is a paucity of endeavors dedicated to preoperative prediction of post-intubation hemodynamic instability (PIHI), despite its critical significance in enhancing the perioperative anesthesia quality and safety, as well as mitigating postoperative risks [21].

The patient’s hemodynamic variables exhibit rapid responsiveness in the operative procedure, thereby providing valuable feedback for anesthetists to make appropriate adjustments in subsequent treatment steps, such as drug administration. However, this is not applicable at the time of initial drug infusion, as there is no patient response yet. Following intubation, the patient is at higher risk for hemodynamic instability, and thus, the anesthetist’s empirical determination of the initial dose, based on preoperative influencing factors [22], is critical for ensuring anesthesia safety. Therefore, preoperatively and precisely evaluating the risk of hemodynamic instability to different drug dosages in diverse patient populations holds paramount reference value for anesthesiologists in the context of perioperative care management and closed-loop intravenous drug administration [23].

The aim of this study is to propose a post-intubation anesthetic risk index of hemodynamic instability and develop a machine-learning model for predicting it. To the best of our knowledge, no prior investigation has integrated perioperative hemodynamic parameters into a comprehensive index value. Moreover, with the help of the predictive ability of machine-learning models [24, 25], we hypothesized that we could develop a data-driven approach for preoperatively predicting the proposed index utilizing preoperative patient information and planned initial drug infusion data. The prediction performances of different models were compared, and the contribution of the variables were evaluated in the secondary analysis.

## Materials and methods

### Study sample and data description

This single center retrospective study was approved by the institutional ethics committee of Chinese Academy of Medical Sciences & Peking Union Medical College (I-23PJ746), with waivers for consent. All data underwent de-identification procedures for the purpose of preserving privacy and ensuring data confidentiality. The manuscript adheres to the Strengthening the Reporting of Observational Studies in Epidemiology (STROBE) guidelines for observational studies.

The original dataset comprises data from a cohort of 56,083 adult (age ≥ 18 yr) surgical patients who underwent general anesthesia with intravenous induction and endotracheal intubation at Peking Union Medical College Hospital, collected over a span of 9 years from 2012 to 2020. Given our primary objective of establishing a general framework to evaluate post-intubation hemodynamic instability, independent of specific surgical categories, we selectively reserved the patients with American Society of Anesthesiologists (ASA) scores of 1 and 2. This inclusion criterion was employed to mitigate the disproportionate influence of severe complications of the patients with ASA score 3 or 4 on the model complexity and study outcomes. Furthermore, by excluding the outliers and the data with missing values for key variables, the dataset was refined to encompass a cohort of 23,305 patients, thereby ensuring data integrity and enhance the robustness of subsequent analysis. By leveraging the Electronic Health Record System (EHRS) and Anesthesia Information Management System (AIMS) of the hospital, we retrospectively obtained comprehensive data of the study samples including preoperative patient information, initial drug infusion details, and perioperative hemodynamic profiles.

### Preoperative patient information

The differences in patient characteristics greatly influence the process of the operation and notably affects the patient anesthetic response. A fundamental tenet of feature selection involves prioritizing attributes derived from readily obtainable basic information during each surgical procedure. This approach ensures the applicability of the prediction framework across a wider spectrum of clinical scenarios. The study utilized basic patient information from the EHRS, including age (median = 47 years, range = 18–91 years), sex (0 = female, 1 = male, female proportion 67.39%), height (median = 164 cm, 25th percentile = 160 cm, 75th percentile = 170 cm), weight (median = 64 kg, 25th percentile = 56 kg, 75th percentile = 72 kg), body mass index (BMI) (median = 23.71 kg/m^2^, 25th percentile = 21.45 kg/m^2^, 75th percentile = 26.16 kg/m^2^) and hypertension (0 = no hypertension, 1 = hypertension, hypertension proportion 14.49%). These parameters were considered in the modeling process according to the empirical experience of anesthetist and data availability. Furthermore, the preoperative physiological characteristics of patients, including systolic pressure (SP), diastolic pressure (DP) and heart rate (HR), were retrospectively obtained from the first stable measurements recorded in the AIMS. The median (25th percentile, 75th percentile) values of these preoperative physiological characteristics were SP = 130 (117, 144) mmHg, DP = 78 (70, 86) mmHg, and HR = 78 (69, 88) BPM (beats per minute), respectively.

### Initiatory drug infusion

The initiatory drug infusion, which is the primary pharmacological intervention undertaken by the anesthetist during surgery, constitutes a fundamental step towards achieving favorable anesthesia safety. The collection of initiatory drug infusion data included the dosages of four frequently utilized drugs in general anesthesia, specifically fentanyl, lidocaine, propofol, and rocuronium. The determination of drug dosages is based on the anesthetist’s evaluation of the patient’s clinical status, and is conventionally expressed as the amount of drug administered (in microgram or milligrams) per kilogram (kg) of the patient’s body weight. Table 1 displays the ranges of both absolute and converted drug dosages in the whole dataset, along with their corresponding means and standard deviations. In this study, the converted drug dosages of fentanyl, lidocaine, propofol, and rocuronium are considered as the input features of the model.

**Table 1:** Drug characteristics. The range and converted range of the drug doses are represented as (minimum, maximum). The median (25th percentile, 75th percentile) of the converted doses for different drugs are also provided

Drug	Range of the drug doses (mg)	Range of the converted drug doses (mg/kg)	Median (25th percentile, 75th percentile) of the converted drug doses (mg/kg)
Fentanyl	(50, 500) *1E-3	(0.455, 5.882) *1E-3	1.667 (1.429, 2.000) *1E-3
Lidocaine	(0, 100)	(0.000–2.222)	0.606 (0.471, 0.727)
Propofol	(50, 250)	(0.521–4.902)	2.174 (1.940, 2.500)
Rocuronium	(20, 100)	(0.190–2.174)	0.714 (0.641, 0.800)

The converted drug doses are calculated according to the amount of drug administered (mg) per kilogram (kg) of the patient’s body weight.

### Integrated coefficient of variation

Hemodynamic profiles serve as the primary foundation for constructing the PIHI index. The systolic pressure, diastolic pressure and heart rate were measured six times in 1 minute during each operation and stored in the AIMS, comprising sequences of hemodynamic characteristics. As shown in Eq. 1, by describing the extent of variability concerning the mean value, the coefficient of variation (CV) value were applied to indicate the instability of a sequence. The inherent property of ratio values of the CV serves to eliminate the adverse effects of varying scales and dimensions in different types of sequences. To comprehensively integrate the CV value of different hemodynamic characteristics, we estimate their weights according to Eq. 2. The weight of each hemodynamic parameter is calculated based on the corresponding information entropy [26], which generates a smaller weight for a more homogeneous sequence set. In this study, the CV values captured from the three hemodynamic response sequences were merged to obtain the integrated coefficient of variation (ICV) value as the PIHI index, according to Eq. 3.

1 $${y}_{is}=\frac{\sqrt{\frac{1}{K}\sum \limits_{k=1}^K{\left({x}_{ik}^s-\frac{1}{K}\sum \limits_{k=1}^K{x}_{ik}^s\right)}^2}}{\frac{1}{K}\sum \limits_{k=1}^K{x}_{ik}^s}$$

2 $${w}_s=1+\frac{1}{\log (N)}\sum \limits_{i=1}^N\left[\left({y}_{is}/\sum \limits_{i=1}^N{y}_{is}\right)\times \log \left({y}_{is}/\sum \limits_{i=1}^N{y}_{is}\right)\right]$$

3 $${z}_i=\sum \limits_{s=1}^S\left({w}_s{y}_{is}/\sum \limits_{s=1}^S{w}_s\right)$$

where *y*_*is*_ is the CV value of the *s-th* sequence of patient *i*; ${x}_{ik}^s$ is the *k-th* measured value in the *s-th* sequence of patient *i*; *w*_*s*_ is the overall weight of the *s-th* sequence sets; *z*_*i*_ is the ICV value of patient *i*; *K* represents the sequence length; *N* is the number of patients; and *S* is the number of hemodynamic characteristics, which in this paper is 3.

**Fig. 1:**
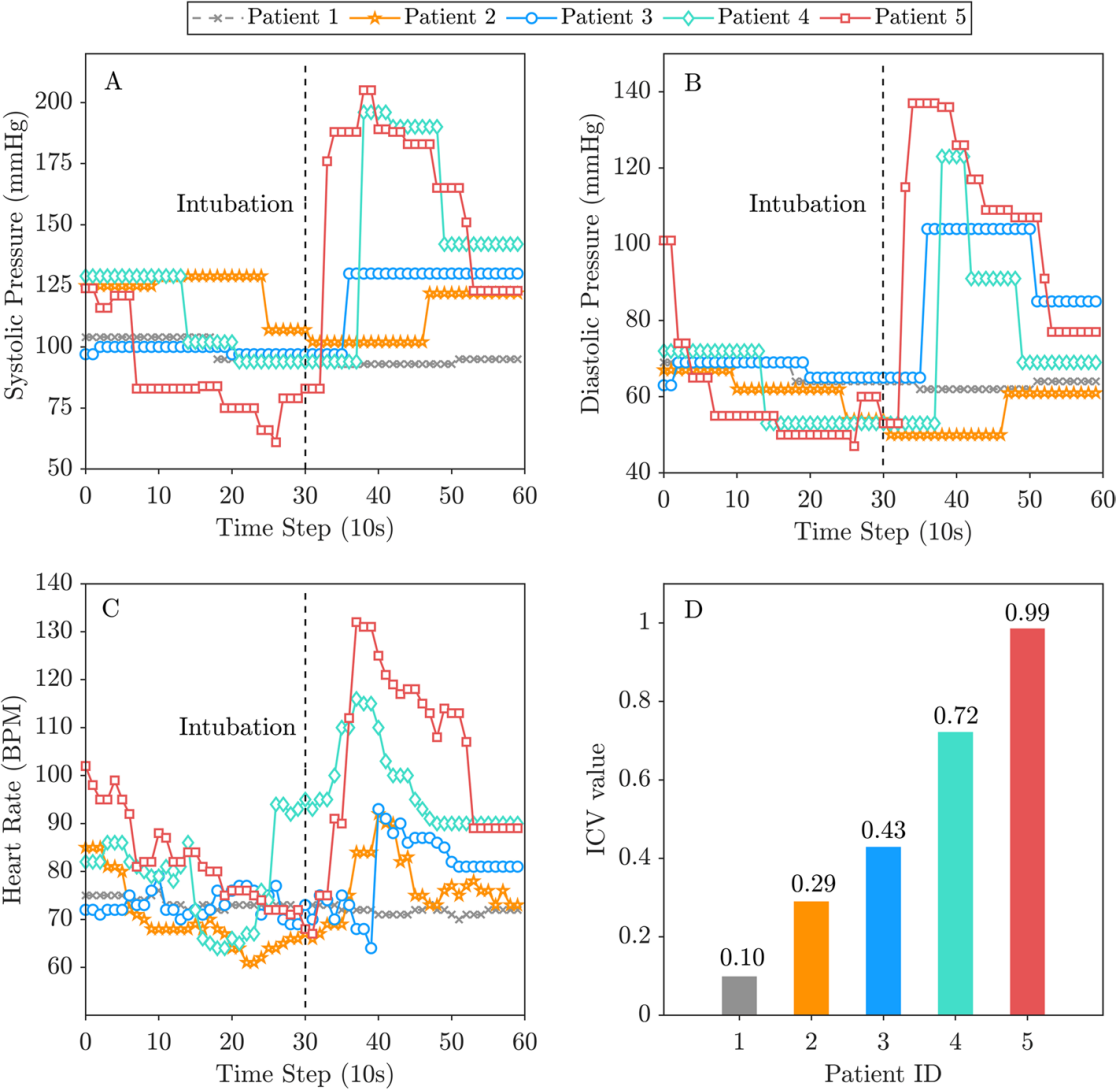
Sequences of hemodynamic characteristics around intubation and the calculated ICV values. Plots were generated by using the anesthesia monitoring data of 5 specific patients. In A, B and C, the sequences of the systolic pressure, diastolic pressure and heart rate of the 5 patients are presented with a time step of 10 seconds. The time of intubation is plotted as a dotted line to distinguish the pre- and postintubation periods. The calculated ICV values of the patients, as the anesthetic risk index of PIHI, are provided in D. *BPM* beats per minute, *ICV* integrated coefficient of variance

In general, a larger ICV value could indicate that the patient may be at higher risk of an unexpected PIHI. The original minimum and maximum values were determined to be 0.0055 and 0.6746, respectively. For convenience, the ICV values are normalized to values between 0 and 1, and proposed to be the anesthetic risk index of PIHI in this study. To depict the whole picture of the sequence around the intubation, 5 minutes of data (around 30 data points) before and after the intubation time are collected and utilized to calculate the ICV index. Figure 1 shows the sequences for different patients and the achieved ICV values. After intubation, patient 5 experienced significant unexpected changes in systolic pressure (Fig. 1A), diastolic pressure (Fig. 1B) and heart rate (Fig. 1C), while comparatively, patient 1 showed more stable anesthetic responses. Figure 1D illustrates a discernible contrast in the ICV value between patient 1, exhibiting a lower ICV value of 0.10, and patient 5, demonstrating a larger ICV value of 0.99 due to the unstable or sudden change of hemodynamic characteristics.

### Statistical analysis

**Fig. 2:**
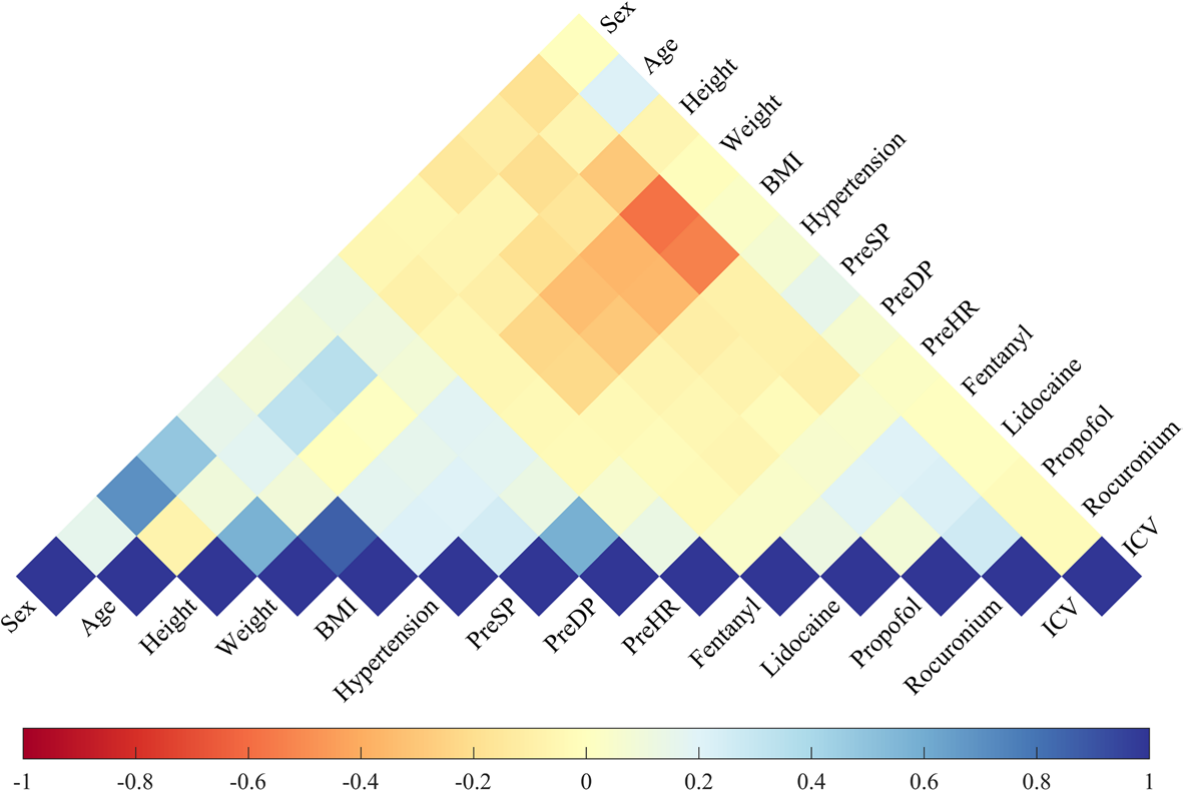
Pearson correlation coefficients among the variables. Different colors are assigned to symbolize the diverse coefficient values. Fentanyl, Lidocaine, Propofol, and Rocuronium represent the converted doses of drugs at initial infusion. *PreSP* preoperative systolic pressure, *PreDP* preoperative diastolic pressure, *PreHR* preoperative heart rate, *BMI* body mass index

A correlation analysis was conducted on the various features of the original data, and their Pearson correlation coefficients are depicted in Fig. 2. It is evident that there exists a certain degree of correlation among the variables, with weight exhibiting the strongest negative correlation with rocuronium, yielding a correlation coefficient of − 0.582 (*p* < 0.01). Additionally, weight demonstrates the highest positive correlation with BMI, with a correlation coefficient of 0.862 (p < 0.01). The proposed ICV index demonstrates a moderate correlation with other variables. Specifically, it displays a relatively higher correlation with age, indicated by a significant Pearson correlation coefficient of 0.204 (p < 0.01).

### Model architecture

**Fig. 3:**
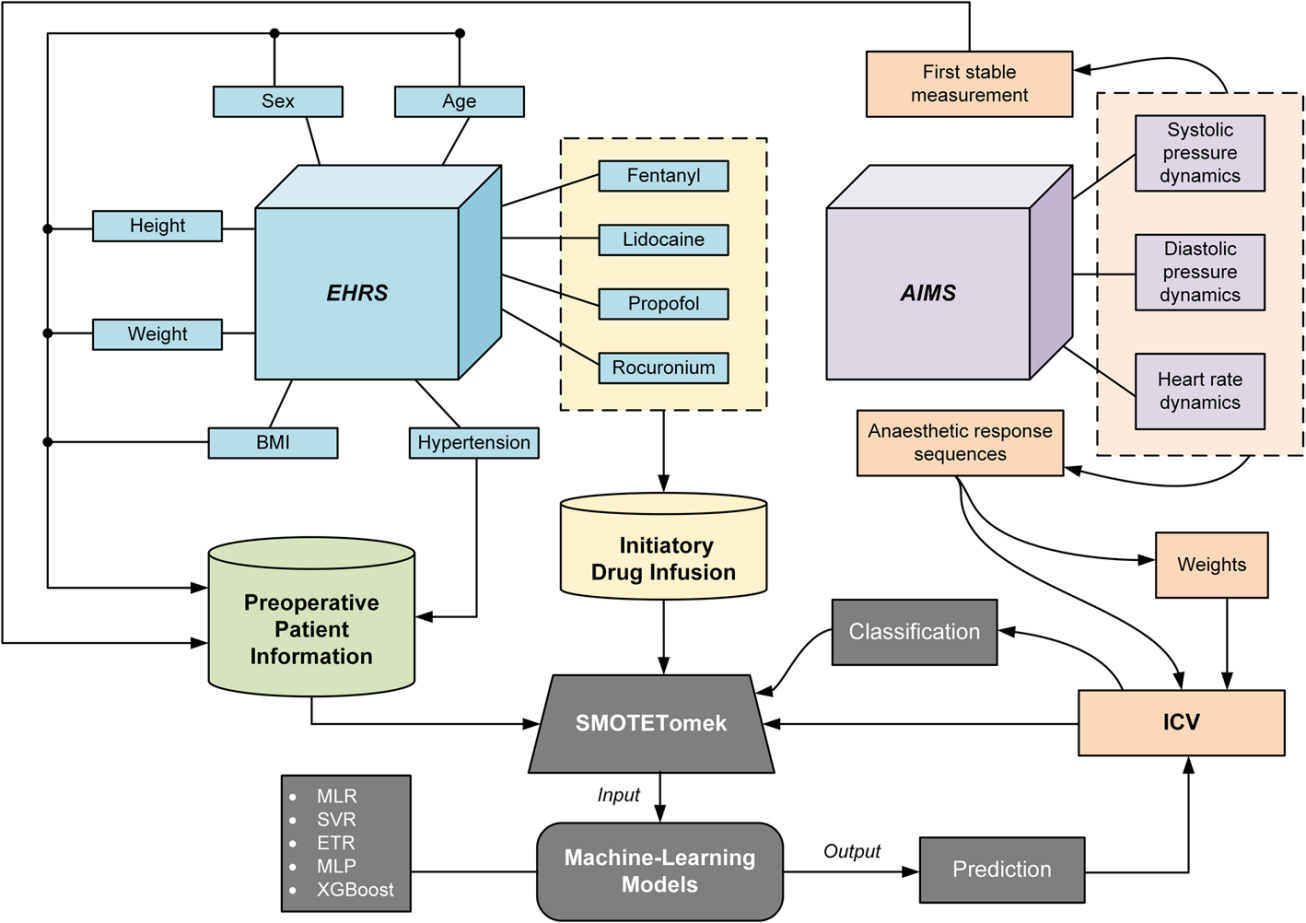
Model architecture with preoperative patient information, perioperative initiatory drug infusion and ICV. *EHRS* electronic health record system, *BMI* body mass index, *AIMS* anesthesia information management system, *MLR* multiple linear regression, *SVR* support vector regression, *ETR* extra tree regression, *MLP* multilayer perceptron, *XGBoost* extreme gradient boosting, *SMOTETomek* synthetic minority over-sampling technique with Tomek links identification, *ICV* integrated coefficient of variance

To deeply understand the nonlinear impact of different variables on post-intubation hemodynamic instability, a machine learning framework was constructed for predicting ICV. The model architecture is given in Fig. 3. The input features of the prediction models consist of preoperative patient information (age, sex, height, weight, BMI, hypertension, preoperative systolic pressure, preoperative diastolic pressure, and preoperative heart rate) and the converted doses for different drugs in initiatory infusion (fentanyl, lidocaine, propofol, and rocuronium), which are extracted from the EHRS and AIMS. The PIHI index, that is the ICV value of the patient, is taken as the output feature in the models. We choose the possible variables that may affect the post-intubation hemodynamic stability according to the collective experience of anesthetist and data availability. For example, the preoperative volemia assessment results are not always available in our dataset and can hardly be applied in the machine-learning training process for building up a more general model. Meanwhile, other comorbidities except for hypertension are not taken into account. Because chronic high blood pressure can lead to increased collagen production in the arterial wall [27], resulting in arterial rigidity and decreased vascular elasticity, which may influence the hemodynamic instability. Even though the hypertension may overlap with preoperative systolic pressure to some extent, the machine learning methods with more complex architecture were recommended to better mitigate multicollinearity [28].

Furthermore, it should be noted that the original dataset is insufficient to facilitate comprehensive model training, owing to the data imbalance pertaining to the predicted ICV value. In light of this, balancing the data distribution could be helpful, e.g., by using oversampling and undersampling methods. Given the certified and widely used techniques for addressing class imbalance, the continuous ICV values are first converted to a typical class imbalance problem. The ICV values are classified into 5 classes, with ranges of [0, 0.2), [0.2, 0.4), [0.4, 0.6), [0.6, 0.8) and [0.8, 1.0]; this establishes a class imbalance in the dataset, with class 1 (10.08%), class 3 (30.29%), class 4 (3.32%) and class 5 (0.16%) as minority classes and class 2 (56.15%) as the majority class. The SMOTETomek (synthetic minority over-sampling technique with Tomek links identification) technique [29] is then utilized to solve this class imbalance problem; SMOTE creates new minority class data by interpolating the adjacent original data in the minority class, and Tomek is used to identify and remove noisy or borderline samples caused by the creation of these new data.

Following the resolution of the imbalanced data issue, the extended dataset was randomly divided into training and testing sets in a 9:1 ratio. The statistics of the input features in the training and testing sets are given in Table 2. We found that the training and testing sets exhibited a comparable distribution, with no significant differences. Ten-fold cross-validation was performed with the training dataset to ensure the robustness of the prediction models by assessing the model performance with averaged evaluation indices over different fold partitions.

**Table 2:** The statistics of the input features in the training and testing sets

Features	Training sets	Testing sets	*P* value ^a^
Sex			
Male (1)	30.17%	29.97%	0.748
Female (0)	69.83%	70.03%	
Age	49.98 (13.88)	50.07 (13.96)	0.611
Height (cm)	164.20 (6.72)	164.22 (6.75)	0.882
Weight (kg)	64.51 (11.32)	64.52 (11.33)	0.922
BMI	23.84 (3.38)	23.84 (3.32)	0.907
Hypertension			
Yes (1)	17.01%	17.64%	0.206
No (0)	82.99%	82.36%	
Pre SP (mmHg)	133.55 (20.41)	133.26 (20.51)	0.282
Pre DP (mmHg)	78.42 (12.12)	78.49 (12.16)	0.624
Pre HR (BPM)	79.06 (13.08)	79.03 (13.12)	0.874
Fentanyl (μg/kg)	1.76 (0.53)	1.77 (0.54)	0.494
Lidocaine (mg/kg)	0.578 (0.248)	0.578 (0.249)	0.894
Propofol (mg/kg)	2.23 (0.45)	2.22 (0.44)	0.732
Rocuronium (mg/kg)	0.730 (0.113)	0.728 (0.113)	0.333

The sex and hypertension are discretized to 0–1 value and presented in percentage; Other continuous features are described by mean value (standard deviation, Std); The training and testing sets exhibited a comparable distribution, with no significant differences according to T-test result.

*Pre* preoperative, *SP* systolic pressure, *DP* diastolic pressure, *HR* heart rate, *BMI* body mass index, *BPM* beats per minute.

^a^*P* value of T-test between the training and testing sets

### Machine learning models

Five typical machine learning models were created to predict the ICV value using multiple linear regression (MLR), support vector regression (SVR), extra tree regression (ETR), multilayer perceptron neural network (MLP), and eXtreme Gradient Boosting (XGBoost) regression methods. MLR was used as a baseline method, as it establishes a linear relationship between the input features and output features. The SVR method produces a classical model that provides a nonlinear solution by mapping input features into a higher-dimensional feature space [30]. As the most commonly used mapping kernel, the radial basis function (RBF) was used in this work to establish the SVR model. ETR [31] is an extension of random forest regression and has been shown to be more reliable than random forest regression in terms of resisting overfitting [32]. MLP is a typical artificial neural network with multiple hidden layers and neuron units and is mainly trained by the backpropagation algorithm [33]. The network parameters were adjusted and updated through iterative computation of their gradients, that is, partial derivative calculations. Finally, XGBoost is a method that operates under the gradient boosting framework [34]. Its decision tree ensembles are composed of sequentially additive trees that learn the residual errors of predictions. All these models were implemented using the mature packages in Python 3.7 [35, 36].

### Model hyperparameters

Grid search and cross-validation were adopted to determine the model hyperparameters. The SVR model was trained with RBF kernel coefficient of 100 and a penalty parameter of 1.5 (L2 penalty) to ensure robust model regularization. The optimal ETR model was trained with 890 estimators and without assigning a maximum tree depth. Two hidden layers constructed by 66 and 65 neuron units were set for the MLP model with the Relu activation function. The learning rate and momentum coefficient for the MLP model were 0.001 and 0.7, respectively. The optimal hyperparameters of the XGBoost model were identified with 648 estimators, a maximum tree depth of 16 and a minimum child weight of 9.

### Performance metrics

The mean absolute error (MAE), root mean square error (RMSE), mean absolute percentage error (MAPE) and R-squared index (R^2^) were used to evaluate the prediction performance of the different models based on Eqs. 4–7.

4 $$MAE=\frac{1}{n}\sum \limits_{i=1}^n\left|{\hat{y}}_i-{y}_i\right|$$

5 $$RMSE=\sqrt{\frac{1}{n}\sum \limits_{i=1}^n{\left({\hat{y}}_i-{y}_i\right)}^2}$$

6 $$MAPE=\frac{100\%}{n}\sum \limits_{i=1}^n\left|\frac{{\hat{y}}_i-{y}_i}{y_i}\right|$$

7 $${R}^2=1-\frac{\sum \limits_{i=1}^n{\left({\hat{y}}_i-{y}_i\right)}^2}{\sum \limits_{i=1}^n{\left(\overline{y}-{y}_i\right)}^2}$$

in which ${\hat{y}}_i$ and *y*_*i*_ are the predicted and observed ICV values of the anesthetic responses, respectively, of patient *i*; *n* is the number of patients; and $\overline{y}$ is the average ICV value of the anesthetic responses.

## Results

### Feature difference analysis

**Fig. 4:**
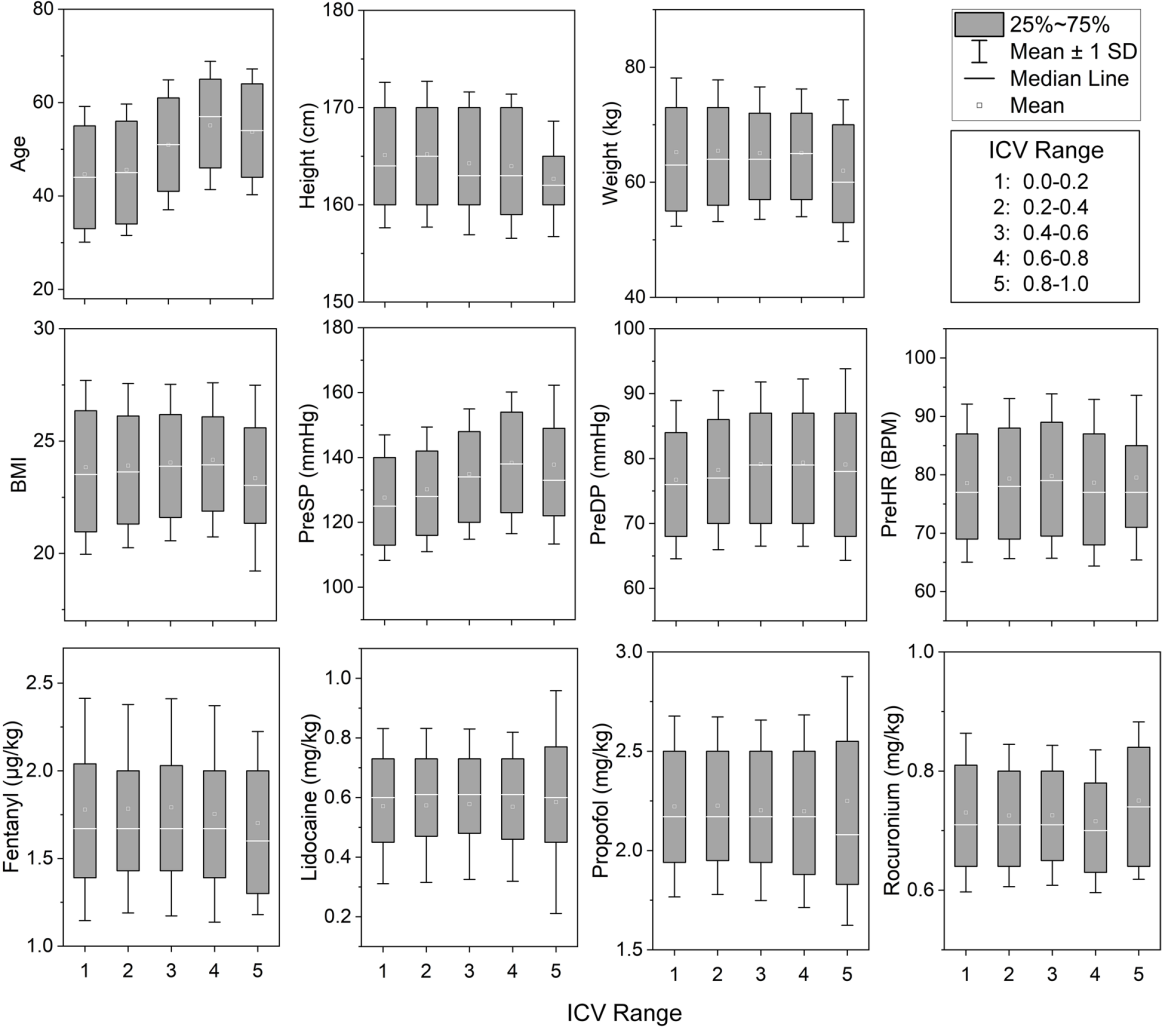
The statistics of the continuous features within the different ICV ranges. Fentanyl, Lidocaine, Propofol, and Rocuronium represent the converted doses of drugs at initial infusion. *PreSP* preoperative systolic pressure, *PreDP* preoperative diastolic pressure, *PreHR* preoperative heart rate, *BMI* body mass index, *BPM* beats per minute, *SD* standard deviation

To assess the differences in feature values associated with post-intubation hemodynamic instability (PIHI), the ICV values were categorized into 5 classes, with ranges of [0, 0.2), [0.2, 0.4), [0.4, 0.6), [0.6, 0.8) and [0.8, 1.0]. Utilizing ANOVA analysis, as detailed in Table 3, it was observed that none of the features satisfied the assumption of equal variances based on Levene’s test. Consequently, the Welch variance and Brown-Forsythe variance were employed for further analysis. Notably, the features of age, height, BMI, hypertension, preoperative systolic pressure, preoperative diastolic pressure, preoperative heart rate, as well as the initial doses of propofol and rocuronium exhibited significant differences (*P* < 0.05) across the various ICV ranges. Refer to Fig. 4 for a graphical representation of the statistical distribution of continuous features within the different ICV ranges.

**Table 3:** The feature difference analysis for different ICV ranges

Features	Levene Statistic (*P* value)	Welch Statistic (*P* value)	Brown-Forsythe Statistic (*P* value)
Sex	5.591 (0.000)	1.277 (0.279)	1.282 (0.276)
Age	3.860 (0.004)	248.949 (0.000)	252.249 (0.000)
Height (cm)	2.624 (0.033)	23.075 (0.000)	24.891 (0.000)
Weight (kg)	18.782 (0.000)	2.146 (0.075)	2.155 (0.073)
BMI	12.310 (0.000)	3.202 (0.014)	2.986 (0.019)
Hypertension	102.908 (0.000)	24.034 (0.000)	22.978 (0.000)
Pre SP (mmHg)	11.700 (0.000)	108.014 (0.000)	95.847 (0.000)
Pre DP (mmHg)	3.729 (0.005)	18.817 (0.000)	16.912 (0.000)
Pre HR (BPM)	2.645 (0.032)	4.003 (0.004)	3.950 (0.004)
Fentanyl (μg/kg)	3.905 (0.004)	0.969 (0.425)	0.977 (0.419)
Lidocaine (mg/kg)	4.112 (0.002)	0.524 (0.718)	0.414 (0.798)
Propofol (mg/kg)	4.828 (0.001)	3.410 (0.010)	2.759 (0.028)
Rocuronium (mg/kg)	8.808 (0.000)	2.470 (0.045)	2.495 (0.042)

The sex and hypertension are discretized to 0–1 value; Welch variance and Brown-Forsythe variance are utilized as all features failed to pass the Levene’s test for equality of variances.

*Pre* preoperative, *SP* systolic pressure, *DP* diastolic pressure, *HR* heart rate, *BMI* body mass index, *BPM* beats per minute.

### Evaluation of ICV effectiveness

The weights of the hemodynamic response sequences are achieved for designing objective and reasonable structure of the ICV. As mentioned above, three hemodynamic response sequences, including systolic pressure (SP) sequence, diastolic pressure (DP) sequence and heart rate (HR) sequence, were utilized to describe the hemodynamic instability after intubation. To calculate the ICV value, the weight of each hemodynamic response sequence is obtained by measuring variable homogeneity according to Eq. 2. After normalization, the weights of the SP, DP and HR sequences were 0.342, 0.355 and 0.303, respectively. This result helps to take a look into the data-driven structure of the ICV. For the cases in this study, the variability extent of SP, DP and HR fluctuation among the populations are basically at the same level, while with DP higher (0.355), SP moderate (0.342) and HR lower (0.303), indicating their significances in constructing the risk index. The weights may exhibit slight variability in the event of alterations to the distributions of SP, DP, and HR values across populations.

**Fig. 5:**
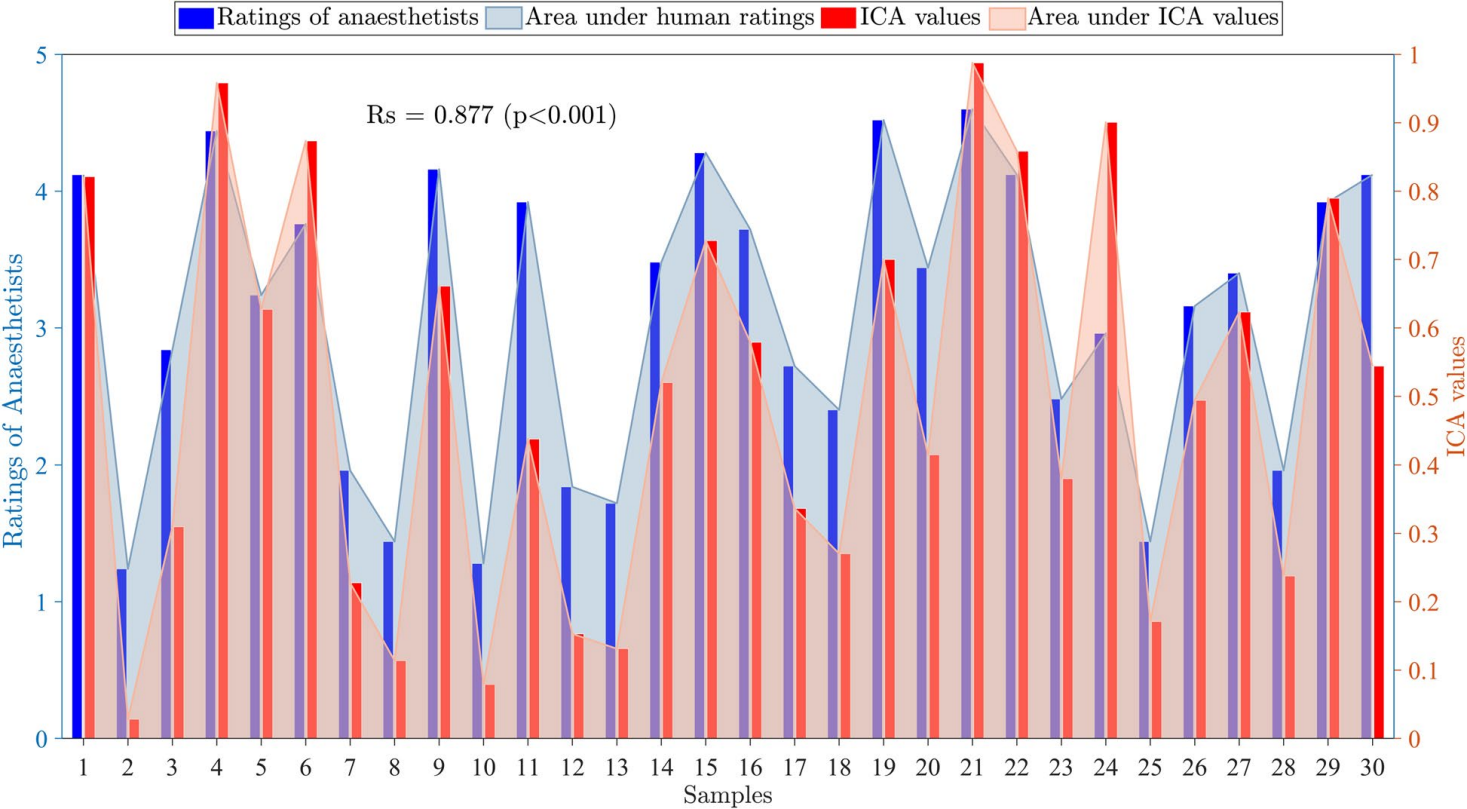
The average ratings of anesthetists and the computed ICA values. The ICA values are consistent with the human ratings, with Spearman correlation coefficient of 0.877 (*P* < 0.001). *Rs* Spearman correlation coefficient, *ICV* integrated coefficient of variance

In order to validate the effectiveness of the proposed ICV value in quantifying hemodynamic instability, we randomly selected 30 data samples of the hemodynamic response sequences for a questionnaire. Twenty-five expert anesthetists were asked to rate the plotted samples of the sequences on a scale ranging from level 1 (indicating stability) to level 5 (indicating instability). The average ratings provided by the anesthetists and the computed ICA values are depicted in Fig. 5. It is found that the ICA values are consistent with the human ratings, with Spearman correlation coefficient of 0.877 (*P* < 0.001), affirming the indicator’s capability to accurately capture the level of instability.

### Machine learning model performance

The prediction performance of different machine learning models was evaluated for picking out the most fitted one. Using appropriate models, the ICV values which reflect the post-intubation anesthetic risk of hemodynamic instability, could be predicted accordingly. Table 4 shows a detailed comparison of the performance of the different models on the whole testing datasets according to the MAE, RMSE, MAPE and R^2^ with their corresponding confidence intervals (CIs). It is noted that the ETR and XGBoost models follow the observed data better than the MLR, SVR and MLP models. The ETR model achieved the smallest MAE (0.0512, 95% CI 0.0511–0.0513), RMSE (0.0792, 95% CI 0.0790–0.0794), and MAPE (0.2086, 95% CI 0.2077–0.2095) and the largest R^2^ (0.9047, 95% CI 0.9043–0.9052). In other words, the ETR model outperformed the other models, achieving higher accuracy and reliability.

**Table 4:** Performance of the machine learning models on the testing datasets

Models	MAE (CI)	RMSE (CI)	MAPE (CI)	R^2^ (CI)
MLR	0.2042 (0.2042–0.2042)	0.2414 (0.2414–0.2414)	0.7451 (0.7446–0.7456)	0.1151 (0.1150–0.1151)
SVR	0.0960 (0.0958–0.0963)	0.1068 (0.1066–0.1070)	0.3289 (0.3279–0.3299)	0.8067 (0.8260–0.8274)
ETR	**0.0512** **(0.0511–0.0513)**	**0.0792** **(0.0790–0.0794)**	**0.2086** **(0.2077–0.2095)**	**0.9047** **(0.9043–0.9052)**
MLP	0.1212 (0.1180–0.1243)	0.1546 (0.1522–0.1571)	0.4634 (0.4511–0.4758)	0.6366 (0.6251–0.6482)
XGBoost	0.0590 (0.0588–0.0593)	0.0914 (0.0910–0.0918)	0.2263 (0.2250–0.2276)	0.8731 (0.8720–0.8742)

Performance metrics are presented with 95% confidence intervals.

*MLR* multiple linear regression, *SVR* support vector regression, *ETR* extra tree regression, *MLP* multilayer perceptron, *XGBoost* extreme gradient boosting, *MAE* mean absolute error, *RMSE* root mean square error, *MAPE* mean absolute percentage error, *R*^*2*^ R-squared index, *CI* confidence interval.

Bold values represent the best performance, indicating that the ETR model outperformed the other models with higher accuracy and reliability.

**Fig. 6:**
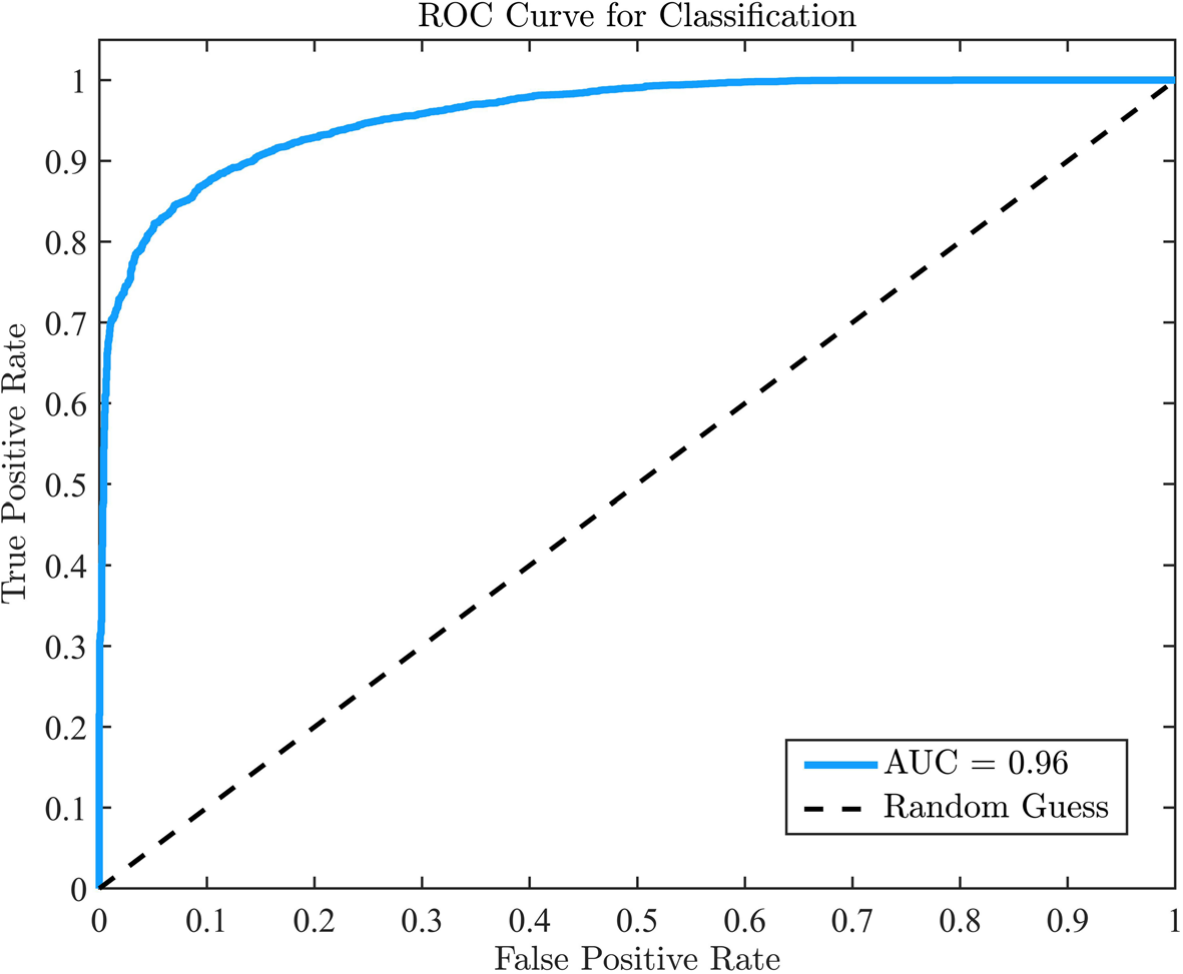
ROC curve of ETR prediction model for classification on ICV values. The AUC value exceeded 0.95, indicating a substantial improvement over random guess. Larger AUC value close to 1 represent better classification performance. *ROC* receiver operating characteristic, *AUC* area under curve

To clearly distinguish the hemodynamic instability and provide simplified instruction to anesthetist, we further classify the ICV values using 0.3 as threshold into stable and instable classes (stable: [0.0, 0.3); instable: [0.3, 1.0]). Figure 6 shows the receiver operating characteristic (ROC) curve of the ETR prediction model employed for the classification of hemodynamic instability. The achieved area under curve (AUC) value of ROC reaches 0.958 (95% CI 0.954–0.962), indicating a substantial improvement over random guess. Notably, the model exhibited impressive performance both in prediction and classification, satisfying the requirement of heightened accuracy for decreasing anesthetic risk involved.

### Feature importance

**Fig. 7:**
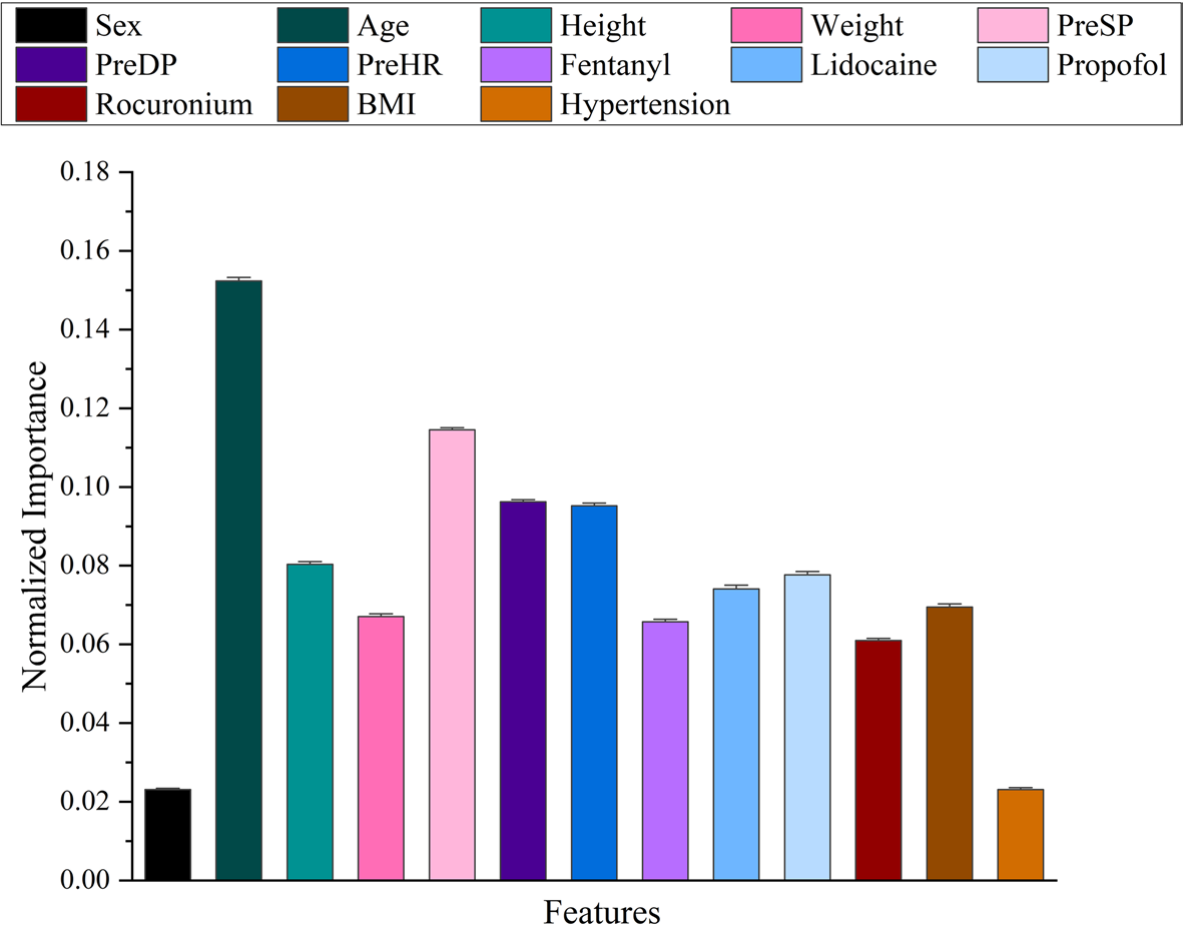
Feature importance of 13 features in the ETR model. The importance values of the features sum to 1 with higher value indicating more important feature. The error bars represent the double standard deviation of the importance values obtained from 10-fold cross-validation. Fentanyl, Lidocaine, Propofol, and Rocuronium represent the converted doses of drugs at initial infusion. *PreSP* preoperative systolic pressure, *PreDP* preoperative diastolic pressure, *PreHR* preoperative heart rate, *BMI* body mass index

The model was further interpreted to get insight on the feature importance, which could tell how the considered input features affect the anesthetic risk. Based on the computation results of ETR model, the Gini importance of the different features can be extracted. Figure 7 shows the feature importance of the ETR model, with higher value indicating more important feature; according to the figure, age, preoperative systolic pressure, preoperative diastolic pressure and preoperative heart rate are more important for accurately predicting the post-intubation anesthetic risk index of hemodynamic instability, with normalized importance value of 0.1524, 0.1145, 0.0962 and 0.0952 respectively.

## Discussion

In this study, we proposed the ICV index as a means of quantifying the patient post-intubation hemodynamic instability (PIHI). The inner structure of the ICV was objectively designed through analyzing the hemodynamic instability on systolic pressure, diastolic pressure and heart rate sequences. Furthermore, we conducted a comparative analysis of various types of machine-learning models to predict the ICV index. The ETR prediction model performed best on the given dataset and was proved to be effective in accuracy, reliability and interpretability.

This retrospective study makes two primary contributions that address the existing gaps in the domain of perioperative anesthesia quality and safety. One contribution is the adoption of the ICV as a PIHI index by merging the various hemodynamic changes after intubation. In previous literatures, most of the attentions were focused on the postoperative outcomes [6–8, 13, 16–18, 20] and perioperative binary judgement on specific symptom using classification models [10, 11, 15, 19]. However, few studies have attempted to quantify the post-intubation anesthetic risk with continuous value and build up the prediction model. Traditionally, the assessment of potential post-intubation risk relies upon the clinical expertise of the anesthetist. The ICV index, which ranges from 0 to 1, provides an opportunity to quantify how a patient physiologically reacts to the intubation under general anesthesia with a specific initial drug infusion. In other words, it could quantitatively and properly describe the extent to which the patient hemodynamic status could be changed during the operation, thereby providing the perioperative anesthetic risk reference.

Another contribution of this study regards the construction of the predictive models and knowledge discovery of the complex anesthetic response system. Machine learning models typically involve a ‘black box’ problem, in which the model performs its function without providing insight into the decision-making process [37]. Additionally, a pressing problem for anesthetists is understanding how the preoperative patient status could affect the perioperative quality [38] and the role of the different drugs in accomplishing their perioperative goals [39]. The knowledge on different importance degrees of the model features implies what factors should be greatly concerned in what order of priority to prevent possible anesthetic risk from data-driven empirical perspective. From this perspective, the ensemble learning methods (ETR and XGBoost) not only presented better prediction accuracy than SVR and MLP but could also help to interpret the variable importance, helping to shed a light on understanding the ‘black box’. As discovered in this study, the feature of age presents evident importance for estimating the patient PIHI; the features of preoperative hemodynamic characteristics, including SP, DP and HR, also show considerable impacts. The availability of other preoperative parameters throughout the dataset would be beneficial to construct a more specific model and achieve a more comprehensive parameter importance evaluation in future works. In the generally available measures, the preoperative physiological status of patient can be deemed as partially controllable characteristic and could be readjusted accordingly with the appropriate instruction; comparatively, as the fully controllable feature, the drug dosage of initial infusion manifests appreciable importance and can be properly manipulated by anesthetists. The medicinal effectiveness of propofol and lidocaine on attenuating hemodynamic responses to intubation/extubation have been noted in the previous researches [40–43]. Other drugs’ influence could be further considered with the current model architecture on an extended dataset.

Furthermore, the study also provides insights on the development of individualized and precise anesthetic treatments. The proper manual evaluation of a professional anesthetist is highly dependent on his/her empirical experience and the comprehensiveness of the given information. From the perspective of artificial intelligence, the proposed method is learning the rich empirical experience of many anesthetists from the readily obtainable basic information of a large number of surgeries. Comparatively, one anesthetist’s experience is limited, which may compromise the evaluation. An erroneous decision has the potential to elicit abrupt and severe hemodynamic changes in the patient, necessitating repeated interventions and resulting in an unstable physiological workload. Facing to each individual patient with unique characteristics, the expertise of a lone anesthetist is inherently constrained, thereby potentially compromising the evaluation process and leading to unfavorable treatment outcomes. To address this concern, the proposed machine-learning model could help anesthetist accurately comprehending the possible perioperative anesthetic risk and taking rational treatment accordingly.

In practical, the constructed machine-learning model could be applied before an operation to verify whether the assumed initial infused drugs are appropriate for the current patient. The prediction could greatly help anesthetists manage the perioperative anesthesia quality and safety right from the start. Additionally, better perioperative hemodynamic profiles may influence postoperative pain [44] and adverse outcomes [45] over the long term, so future studies could investigate the subsequent impacts. For example, the proposed index could be further utilized to predict major adverse cardiovascular events (MACEs) with estimated metabolic equivalents (METs) [46]. The model could also be used to train inexperienced anesthetists, since the wide variability in the characteristics of the patients and the anesthetic drugs doses increase the difficulty of assessing the post-intubation hemodynamic instability. It would be meaningful to fully utilize historical big data to guide inexperienced anesthetists in a substantially shorter duration of time, which is one of the original intentions of this retrospective research. Furthermore, it would be worthwhile to pursue further studies on individualized medication in closed-loop intravenous drug administration by analyzing the heterogeneous hemodynamic responses observed during subsequent infusions. Follow-up studies could also consider the potential impact of patient’s higher ASA scores (i.e., 3 or 4) and the varying levels of expertise among anesthetists on the perioperative anesthesia quality and safety.

## Conclusion

In conclusion, our results suggest that machine learning models are able to preoperatively and precisely predict the post-intubation hemodynamic instability based on the preoperative patient information and planned initial drug infusion data. The ETR model performs best and indicates the varied influence of the different variables on predicting ICV values. Further investigations are warranted to explore more influencing factors in the model, assess the subsequent impacts of the extended models, evaluate its training performance for inexperienced anesthetists and examine the model feasibility in practical implementation of closed-loop intravenous drug administration.

## Data Availability

The datasets used and/or analysed during the current study available from the corresponding author on reasonable request.

## Abbreviations

AIMS: Anesthesia information management system
ASA: American Society of Anesthesiologists
AUC: Area under curve
BMI: Body mass index
BPM: Beats per minute
CI: Confidence interval
CV: Coefficient of variation
DP: Diastolic pressure
EHRS: Electronic health record system
ETR: Extra tree regression
HR: Heart rate
ICV: Integrated coefficient of variation
MAE: Mean absolute error
MAPE: Mean absolute percentage error
MLP: Multilayer perceptron neural network
MLR: Multiple linear regression
RMSE: Root mean square error
ROC: Receiver operating characteristic
R^2^: R-squared index
SMOTETomek: Synthetic minority over-sampling technique with Tomek links identification
SP: Systolic pressure
STROBE: Strengthening the reporting of observational studies in epidemiology
SVR: Support vector regression
XGBoost: Extreme gradient boosting
